# Supplementary material for: Defense Mechanisms Induced by Celery Seed Essential Oil against Powdery Mildew Incited by Podosphaera fusca in Cucumber
Source: J Fungi (Basel). 2023 Dec 27;10(1):17. doi: 10.3390/jof10010017 (PMC10817264; doi:10.3390/jof10010017)
Supplement: Supplementary file 1 [file jof-10-00017-s001.zip › Table S1.pdf]

Table S1. Two-way analysis of variance of the effect of treatment with different concentrations of CSEO (100, 200, and 400 μg mL^{-1}) before and after inoculation of cucumber seedlings with cucumber powdery mildew on disease severity (%) compared to non-treated control sprayed with only dw containing 1 mL L^{-1} of Tween 20.

**Variables**	**DFs[a]**	**Disease severity**	
		**Mean of squares**	**F value**
Sampling time	1	46.9	0.820
Concentrations	3	2861.3***	50.024
Sampling time× Concentrations	3	81.6	1.426
Residuals	24	57.2	

[a] DF= Degree of freedom; *** $P \leq 0.001$.
