# Supplementary material for: Defense Mechanisms Induced by Celery Seed Essential Oil against Powdery Mildew Incited by Podosphaera fusca in Cucumber
Source: J Fungi (Basel). 2023 Dec 27;10(1):17. doi: 10.3390/jof10010017 (PMC10817264; doi:10.3390/jof10010017)
Supplement: Supplementary file 1 [file jof-10-00017-s001.zip › Table S2.pdf]

Table S2. Two-way analysis of variance of the effect of treatment with CSEO (400 µg mL^{-1}) on content of chlorophyll, phenols and flavonoids in cucumber leaves at different time intervals (0, 1, 2, 4, and 8 d) after the treatment.

Variables	DF[a]	Chlorophyll concentration		Phenolic compounds		Flavonoid compounds	
		MS[b]	F[c]	MS	F	MS	F
Set treatment	3	2.7720***	590173	0.15054***	15474.7	0.10185***	454.04
Sampling time	4	0.3412***	72650	0.0011***	113.8	0.07281***	324.56
Treatment× Sampling time	12	0.1179***	25105	0.00527***	541.3	0.02174***	96.92
Residuals	60	0		0.00001		0.00022	

[a]DF=Degree of freedom, [b]MS= Mean of squares, [c]F= F value; *** $P \leq 0.001$.
