# Supplementary material for: Defense Mechanisms Induced by Celery Seed Essential Oil against Powdery Mildew Incited by Podosphaera fusca in Cucumber
Source: J Fungi (Basel). 2023 Dec 27;10(1):17. doi: 10.3390/jof10010017 (PMC10817264; doi:10.3390/jof10010017)
Supplement: Supplementary file 1 [file jof-10-00017-s001.zip › Table S3.pdf]

Table S3. Two-way analysis of variance of the effect of treatment with CSEO (400 μg mL^{-1}) on the activity of β-1,3-glucanase, chitinase, phenylalanine ammonia-lyase, peroxidase and polyphenol oxidase in leaves of cucumber seedlings at different time intervals (0 h, 1, 2, 4, and 8 d) after the treatment.

Variables	DF[a]	β-1,3-glucanase		Chitinase		Peroxidase		Phenylalanine ammonia-lyase		Polyphenol oxidase	
		MS[b]	F[c]	MS	F	MS	F	MS	F	MS	F
Seedling set	3	0.16531***	2725.5	33.36***	2380.31	0.05914***	1841810	308187***	25999.0	0.006780***	9875.60
Sampling time	4	0.07095***	1169.8	1.46***	103.86	0.00670***	208613	28315***	2388.7	0.000425***	618.46
Seedling set × Sampling time	12	0.01281***	211.2	0.52 ***	37.08	0.00134***	41589	4172***	351.9	0.000051***	74.55
Residuals	60	0.00006		0.01		0		12		0.000001	

[a]DF= Degree of freedom, [b]MS= Mean of squares, [c]F= F value; *** $P \leq 0.001$.
