# Supplementary figures and images for: Defense Mechanisms Induced by Celery Seed Essential Oil against Powdery Mildew Incited by Podosphaera fusca in Cucumber
Source: J Fungi (Basel). 2023 Dec 27;10(1):17. doi: 10.3390/jof10010017 (PMC10817264; doi:10.3390/jof10010017)

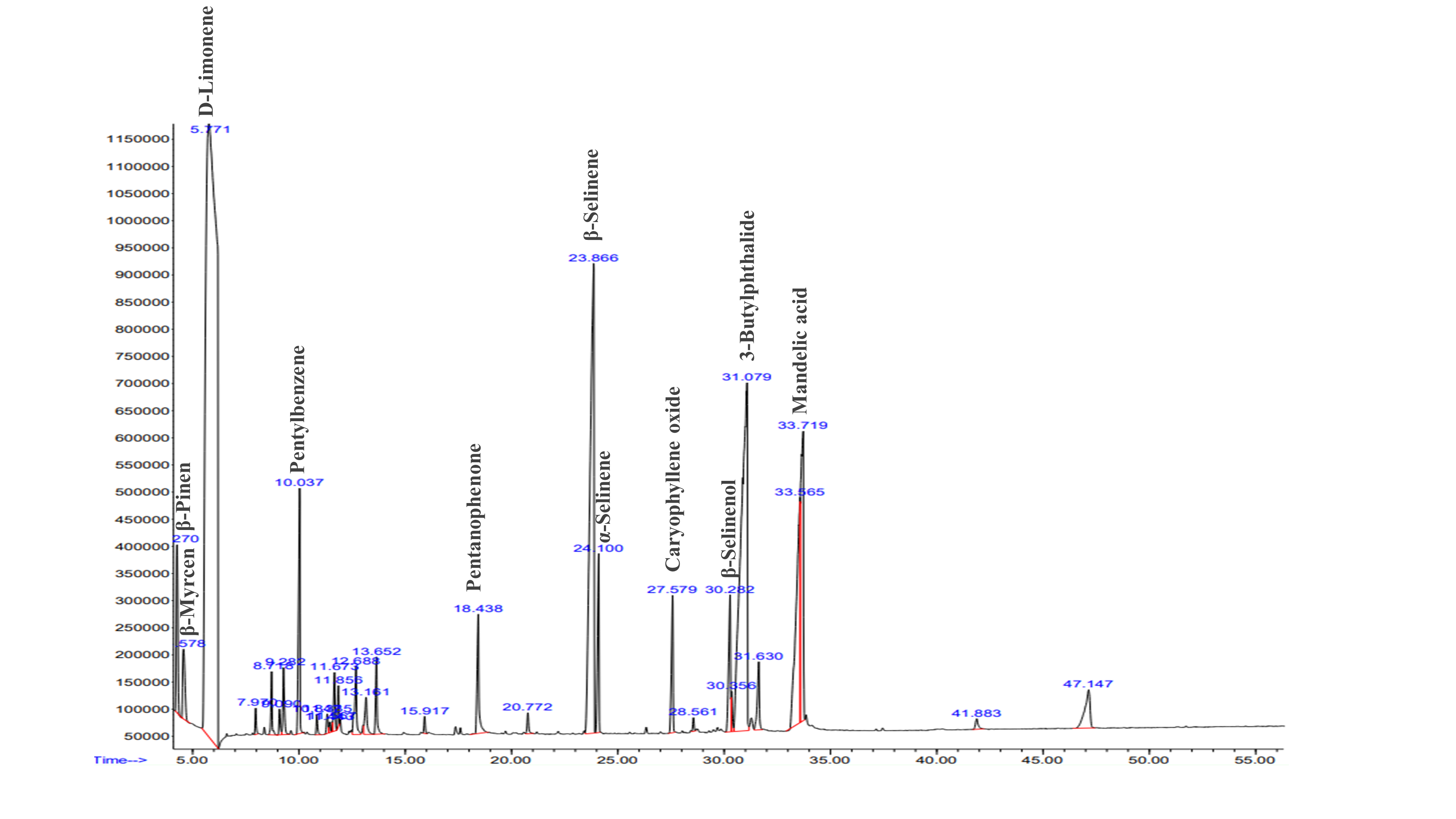

Supplement: Supplementary file 1 [file jof-10-00017-s001.zip › Figure S1.tif]
